# Takotsubo syndrome complicated by left ventricular thrombus and free wall rupture leading to cardiac arrest: a case report of successful life-saving surgical treatment

**DOI:** 10.1093/ehjcr/ytaf601

**Published:** 2025-11-21

**Authors:** Chihiro Morisue, Dai Kawauchi, Kei Yunoki, Noriyuki Tokunaga, Takefumi Oka

**Affiliations:** Department of Clinical Training Center, Tsuyama Chuo Hospital, 1756 Kawasaki, Tsuyama, Okayama 708-0841, Japan; Department of Cardiology, Tsuyama Chuo Hospital, 1756 Kawasaki, Tsuyama, Okayama 708-0841, Japan; Department of Cardiology, Tsuyama Chuo Hospital, 1756 Kawasaki, Tsuyama, Okayama 708-0841, Japan; Department of Cardiovascular Surgery, Tsuyama Chuo Hospital, 1756 Kawasaki, Tsuyama, Okayama 708-0841, Japan; Department of Cardiology, Tsuyama Chuo Hospital, 1756 Kawasaki, Tsuyama, Okayama 708-0841, Japan

**Keywords:** Takotsubo syndrome, Left ventricular thrombus, Cardiac rupture, Cardiac tamponade, Case report

## Abstract

**Background:**

Takotsubo syndrome (TTS) is a stress-induced cardiomyopathy that causes transient left ventricular dysfunction. Although generally considered benign, TTS occasionally leads to serious complications such as cardiac rupture or left ventricular thrombus, both of which carry significant risks.

**Case summary:**

A 69-year-old woman presented to our hospital with dyspnoea after physical and emotional stress. Based on the findings of ST-segment elevation on an electrocardiogram and wall motion abnormality of the left ventricular apex on echocardiography, an acute coronary syndrome was suspected. Emergency coronary angiography showed no significant stenosis in the coronary artery, but left ventriculography showed typical apical ballooning, consistent with TTS. Anticoagulation with heparin was initiated to prevent left ventricular thrombus formation. On the fifth day after admission, echocardiography revealed a left ventricular apical thrombus and slight pericardial effusion, which developed into cardiac tamponade on day 6. While being prepared for emergency surgery, the patient was diagnosed with cardiac rupture and went into cardiac arrest. Therefore, venous-arterial extracorporeal membrane oxygenation was initiated prior to the surgical procedure. Successful repair of the ruptured free wall site in the left ventricular anterolateral region resulted in complete recovery. The patient was discharged on day 35.

**Discussion:**

To our knowledge, this is the first case of TTS complicated by cardiac rupture during anticoagulation treatment for a left ventricular thrombus. Although anticoagulation therapy may have precipitated the bleeding associated with cardiac rupture, the early diagnosis of TTS complications and prompt management, including emergency surgery, may have contributed to the patient’s favourable outcome.

Learning points

- Patients with Takotsubo syndrome (TTS) receiving anticoagulation therapy should be carefully monitored for bleeding complications, including cardiac rupture (CR)
- Early surgical intervention may be important in patients with TTS with CR with haemodynamic instability due to cardiac tamponade

## Introduction

Takotsubo syndrome (TTS) is characterized by transient regional wall motion abnormalities of the left ventricle beyond a single coronary territory and the absence of obstructive coronary artery disease.^1,2^ Although TTS is considered a benign disease, recent studies have shown that TTS has a similar prognosis to acute coronary syndrome, with a higher mortality rate if serious complications occur.^3–5^ Herein, we describe a very rare case in which a patient with TTS developed two potentially fatal complications but survived after surgical treatment.

## Summary figure

**Figure:**
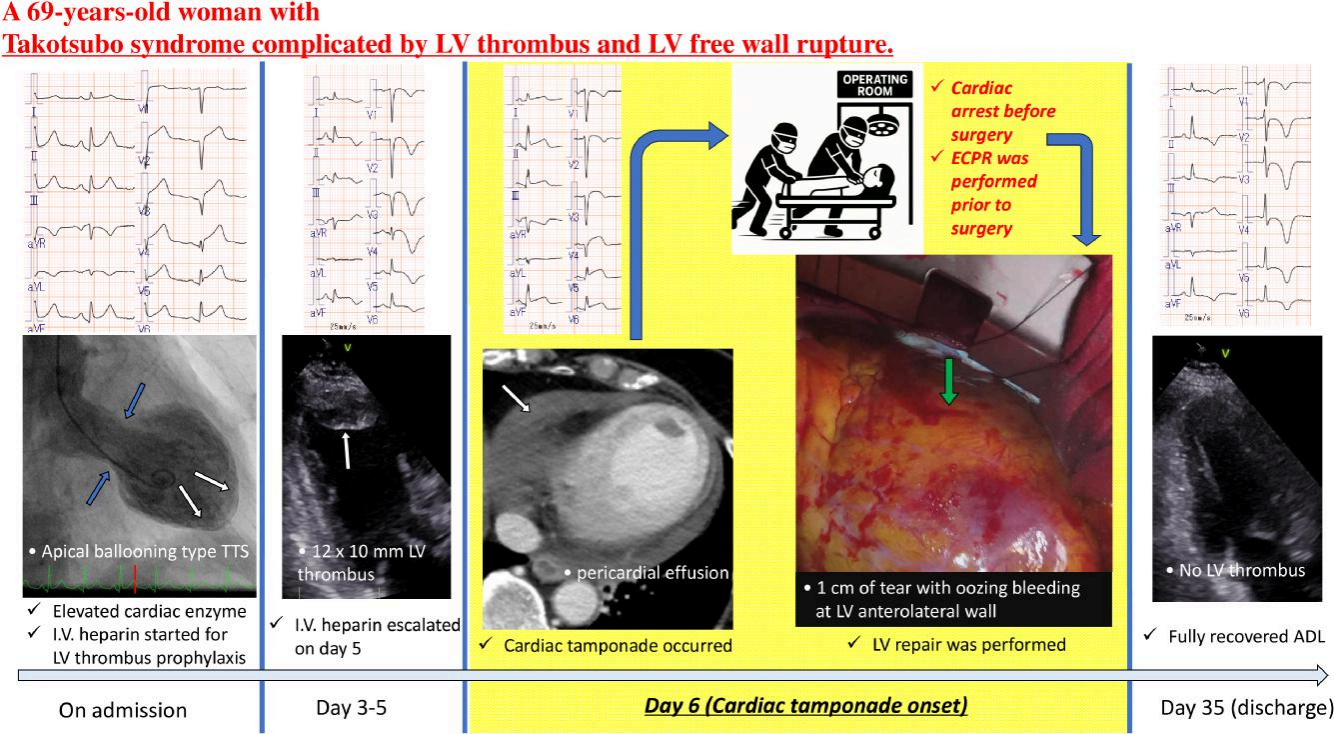


## Case presentation

**Figure 1:**
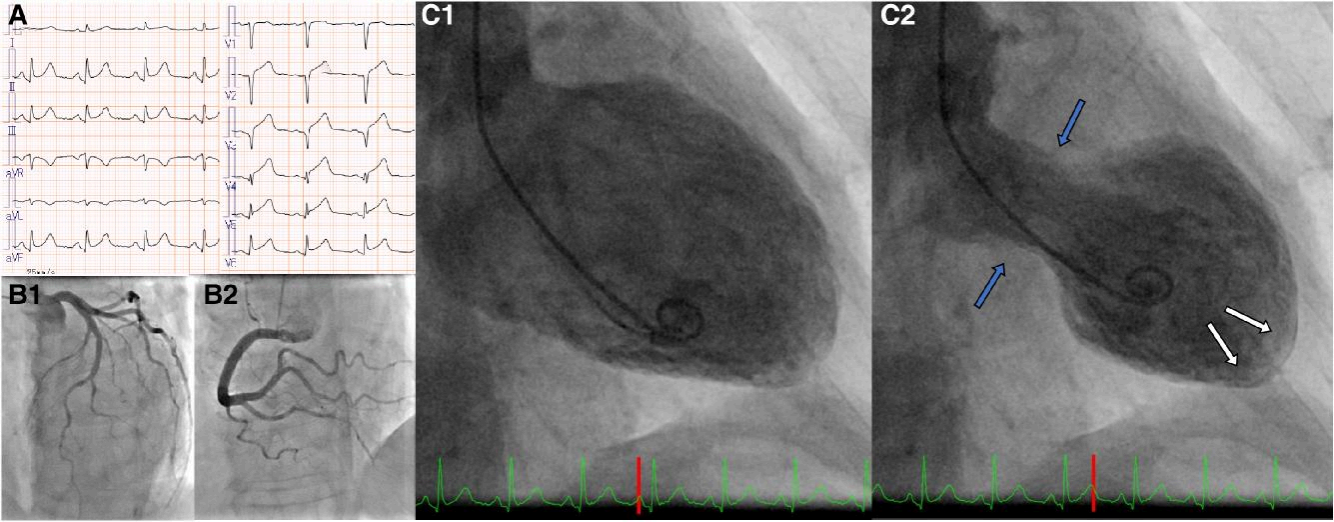
(*A*) The initial electrocardiogram shows ST-segment elevation in leads II, III, aVf, and V2-6. (*B*) Coronary angiography shows no significant stenosis of the coronary arteries. (*C*) Left ventriculography shows typical Takotsubo-like left ventricular wall motion abnormalities with apical ballooning (white arrows) and basal hyperkinesis (blue arrows).

**Figure 2:**
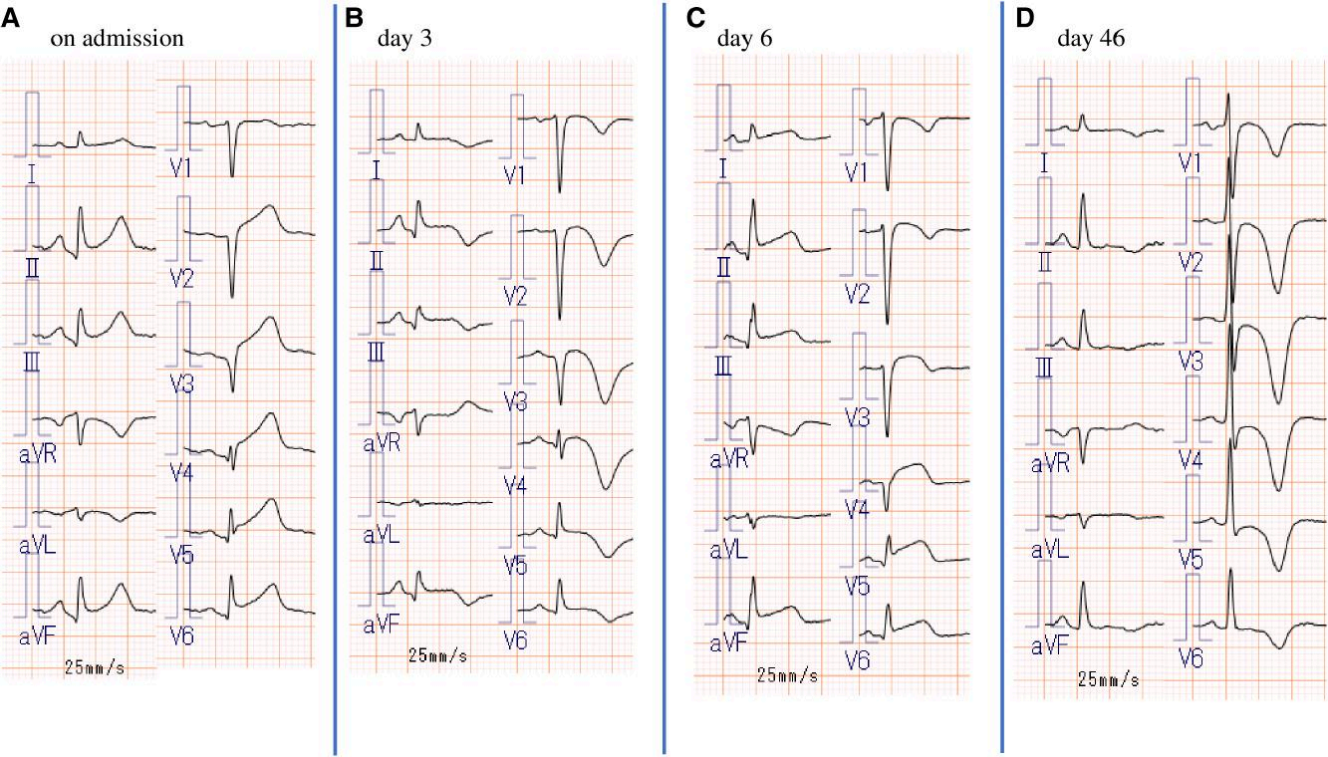
(*A*) The initial electrocardiogram shows ST-segment elevation in leads II, III, aVf, and V2-6. (*B*) Electrocardiogram on day 3 shows inverted T-waves at leads I, II, III, aVF and V1-6. (*C*) Electrocardiogram on day 6 shows ST-segment elevation in leads II, III, aVf and V3-6. (*D*) Outpatient follow-up electrocardiogram on day 46 shows inverted T-waves in leads V1-6.

**Figure 3:**
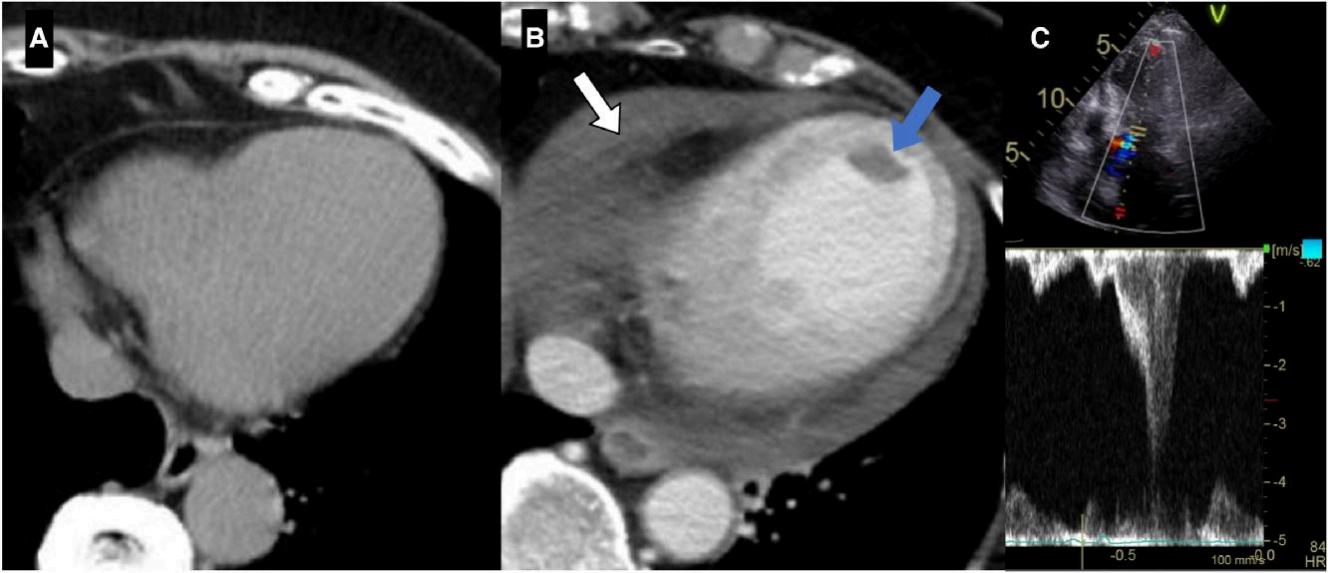
(*A*) Plain computed tomography on admission shows no pericardial effusion. (*B*) Contrast-enhanced computed tomography on day 6 reveals a left ventricular apical thrombus (blue arrow) and moderate pericardial effusion suspected to be haemorrhagic (white arrow). (*C*) Transthoracic echocardiography on day 6 reveals left ventricular outflow tract obstruction with a peak velocity of 4.0 m/s.

**Figure 4:**
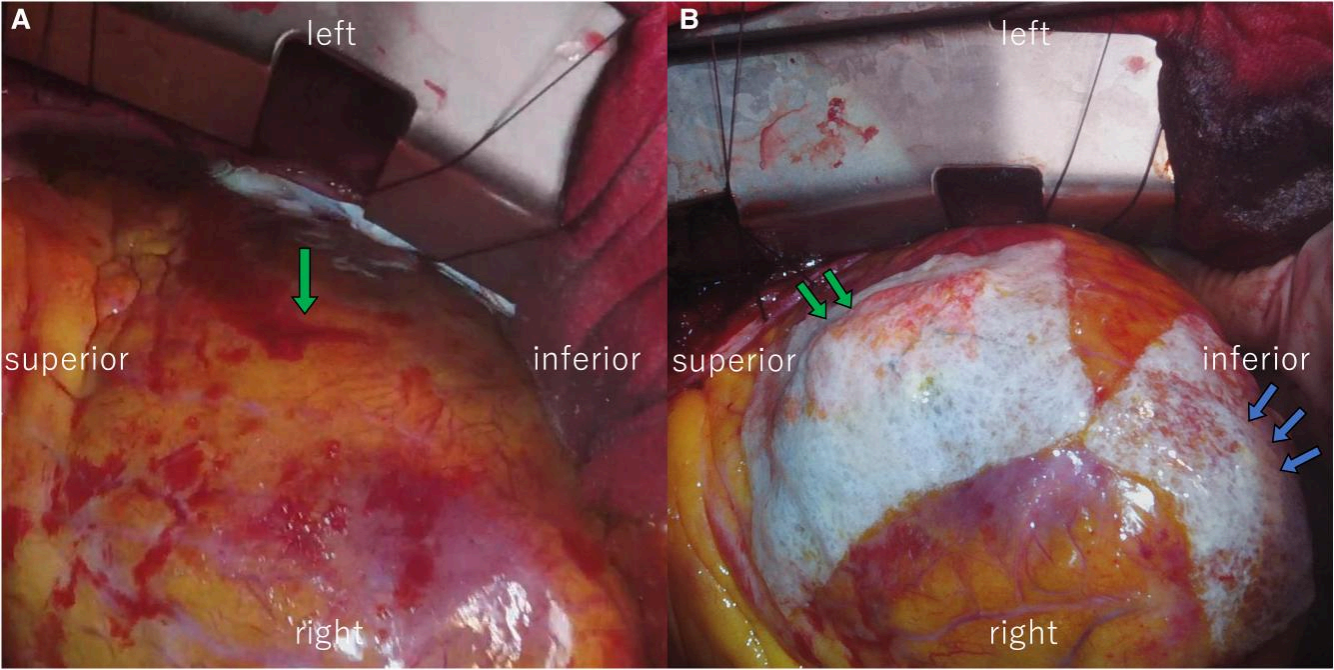
(*A*) Intraoperative findings reveal a 1-cm-long horizontal tear at the left ventricular anterolateral free wall, which is the inflection point for apical ballooning (green arrow). (*B*) The tear is repaired using mattress sutures with two Teflon felt strips and a TachoSil tissue sealing sheet (CSL Behring) (green arrows). The discoloured and fragile left ventricular apex is reinforced (blue arrows).

A 69-year-old healthy woman with no relevant medical history developed sudden dyspnoea. She also reported experiencing physical exhaustion from taking care of her grandchildren for the past few days. On arrival, her consciousness was clear, blood pressure was 101/65 mmHg, heart rate was regular at 80 beats/minute, body temperature was 36.4°C, and oxygen saturation was 98% on room air. There were no significant cardiovascular or respiratory physical findings. A 12-lead electrocardiogram showed a heart rate of 77 beats/minute in sinus rhythm with ST-segment elevation in leads II, III, aVF, and V2-6 (*Figures 1A* and *2A*). Laboratory results are shown in *Table 1*. Transthoracic echocardiography revealed wall motion abnormalities in the left ventricular apical region and no left ventricular outflow tract obstruction (LVOTO) or pericardial effusion. Plain computed tomography also showed no pericardial effusion (*Figure 3A*). Considering the possibility of acute coronary syndrome, emergency coronary angiography was performed and showed intact coronary arteries (*Figure 1B*). Left ventriculography revealed typical TTS-like left ventricular wall motion abnormalities with apical ballooning and basal hyperkinesis (see Supplementary material online, *Video S1, Figure 1C*). She was diagnosed with TTS and started on anticoagulation therapy with intravenous heparin (continuous infusion of 10 000 units per day with a target activated partial thromboplastin time of 1.5–2.0 times the control value) to prevent left ventricular thrombosis. On day 3, a 12-lead electrocardiogram showed inverted T-waves in leads I, II, III, aVF, and V1-6 (*Figure 2B*). The symptoms improved and the clinical course was good until transthoracic echocardiography on day 5 showed a 12 × 10 mm left ventricular apical thrombus and slight pericardial effusion (see Supplementary material online, *Video S2*). Laboratory results revealed an elevated D-dimer concentration of 2.6 µg/mL (normal range: < 1.0 µg/mL). Thus, the anticoagulation therapy was escalated (continuous intravenous heparin infusion of 14 000 units per day with a target activated partial thromboplastin time of 2.0–3.0 times the control value). On day 6, the systolic blood pressure remained low at 80–90 mmHg, and the patient had mild epigastric pain. A 12-lead electrocardiogram showed ST-segment elevation in leads II, III, aVF, and V3-6 (*Figure 2C*). Contrast-enhanced computed tomography revealed a left ventricular apical thrombus and moderate pericardial effusion suspected to be haemorrhagic (*Figure 3B*). Because of suspected CR and haemodynamic instability, intra-aortic balloon pumping was initiated. Transthoracic echocardiography revealed increased pericardial effusion and LVOTO with a peak velocity of 4.0 m/s (*Figure 3C*). The patient was diagnosed with CR associated with TTS. During transfer to the operating room for emergency surgery, she went into cardiac arrest signified by pulseless electrical activity. Extracorporeal cardiopulmonary resuscitation was performed and surgery was initiated. The pericardium was incised and a substantial amount of blood drained from the pericardial cavity (see Supplementary material online, *Video S3*). Intraoperative findings revealed a 1-cm-long horizontal tear in the left ventricular anterolateral free wall, which was the inflection point for apical ballooning (see Supplementary material online, *Video S4, Figure 4A*). The tear was repaired and the discoloured and fragile left ventricular apex was reinforced (*Figure 4B*). Postoperatively, the patient was successfully weaned from venous-arterial extracorporeal membrane oxygenation on day 9 and from intra-aortic balloon pumping on day 10. An angiotensin receptor blocker and beta-blocker were administered on day 10. Transthoracic echocardiography on day 18 showed that the left ventricular apical ballooning and persistent left ventricular apical thrombus were improving with a peak LVOTO velocity of 4.0 m/s (see Supplementary material online, *Video S5*). On day 27, the LVOTO had decreased to a peak velocity of 1.4 m/s with improvement in left ventricular wall motion, and the left ventricular apical thrombus had resolved (see Supplementary material online, *Video S6*). The patient was discharged on day 35 with complete recovery of physical function and no neurological impairment after rehabilitation. At outpatient follow-up on day 46, a 12-lead electrocardiogram showed inverted T-waves in leads V1-6 and improvement in the poor R-wave progression (*Figure 2D*).

**Table 1:** Laboratory data on admission

		normal range
White blood cell (x10^3^/ul)	9.6	3.3–8.6
Basophil (%)	0.5	0.0–3.0
Eosinophil (%)	0.6	0.0–10.0
Neutrophil (%)	83.2	50.0–70.0
Lymphocytes (%)	10.7	30.0–45.0
Monocytes (%)	5.0	2.0–8.0
CRP (mg/dL)	0.13	0.00–0.14
AST (U/L)	23	13–30
ALT (U/L)	15	7–23
BUN (mg/dL)	14.0	8.0–20
Creatinine (mg/dL)	0.67	0.46–0.79
CK (U/L)	200	41–153
CK-MB (IU/L)	26	0–12
hs-TnT (ng/mL)	0.882	<0.100
BNP (pg/mL)	56.9	0–18.4
D-Dimer (µg/mL)	0.5	0.0–1.0

CRP, C-reactive protein concentration; AST, aspartate aminotransferase concentration; ALT, alanine aminotransferase concentration; BUN, blood urea nitrogen concentration; CK, creatine kinase concentration; CK-MB, creatine kinase-MB concentration; hs-TnT, high-sensitivity troponin T concentration; BNP: brain natriuretic peptide concentration.

## Discussion

CR is the most lethal complication of TTS but is very rare, with only 44 reported cases,^6^ while left ventricular thrombus occurs in 2–4% of patients during the acute phase of TTS and can cause systemic embolism.^7,8^ There is only one previous case report of TTS with these two potentially fatal complications.^9^ Compared with the previous case, our case differs in that the left ventricular apical thrombus preceded the CR and the CR was treated surgically rather than conservatively. To the best of our knowledge, this is the first case of a patient surviving cardiac arrest due to CR associated with TTS. The intraoperative video clearly shows the haemorrhagic site of CR (see Supplementary material online, *Video S4*), indicating that surgical intervention was necessary due to haemodynamic compromise.

A left ventricular thrombus in TTS is more frequently detected in female patients with an apical ballooning pattern and elevated troponin concentration.^7^ Patients with TTS are defined as low and high risk by scoring four factors: apical ballooning type, history of vascular disease, left ventricular ejection fraction ≤30% at admission, and first white blood cell count >10 000 cells/µL.^8^ Our patient had several of these factors and was judged to be at high risk of left ventricular thrombosis; thus, prophylactic anticoagulation was administered from the time of admission. Despite anticoagulation therapy, an apical thrombus developed and she had pericardial effusion due to CR at the time of intensification of anticoagulation therapy. During anticoagulation therapy, patients with TTS should be carefully monitored for bleeding complications, including CR.

The risk factors for CR in patients with TTS are female sex, advanced age, persistent ST-segment elevation, high systolic and diastolic pressures, and high left ventricular peak systolic pressure.^10^ These haemodynamic findings in patients with TTS with CR suggest that CR is likely caused by high intracardiac pressure and that LVOTO may also contribute, as confirmed in the present case. Considering that our patient was at high risk of CR, it may have been possible to prevent CR by controlling the blood pressure and LVOTO with beta-blockers from the acute phase. A recent review reported that all patients with TTS with cardiac arrest due to CR died, while many of those without cardiac arrest were saved by surgical intervention.^6^ Although there are reports of conservative treatment in patients with TTS with CR,^6,9,10^ early surgical intervention may be important in cases of haemodynamic instability due to cardiac tamponade.

## Conclusion

To our knowledge, this is the first case of a patient with TTS who developed CR during anticoagulation for a left ventricular thrombus and survived. Although anticoagulation may have precipitated the bleeding associated with CR, the early diagnosis of complications and prompt management, including emergency surgery, may have contributed to the favourable outcome.

## Lead author biography


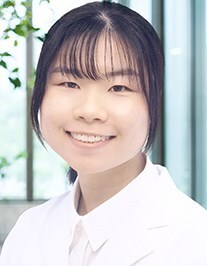


Chihiro Morisue graduated from Okayama University in 2024. She is working as a medical resident in Japan, and interested in internal medicine, anaesthesiology, and intensive care.

## Supplementary Material

ytaf601_Supplementary_Data

## Data Availability

The data underlying this article are available in the article and in the online Supplementary material.
